# Single-cell and spatial analysis reveals the interaction between ITLN1^+^ foam cells and SPP1^+^ macrophages in atherosclerosis

**DOI:** 10.3389/fcvm.2025.1510082

**Published:** 2025-02-13

**Authors:** Ying Li, Shanshan Wang, Ruidan Zhang, Yingying Gong, Yulu Che, Kening Li, Zongfu Pan

**Affiliations:** ^1^ Department of Pharmaceutical Sciences, Institute of Pharmacology, Zhejiang University of Technology, Hangzhou, China; ^2^ Department of Pharmacy, Center for Clinical Pharmacy, Cancer Center, Zhejiang Provincial People’s Hospital (Affiliated People’s Hospital), Hangzhou Medical College, Hangzhou, Zhejiang, China; ^3^ School of Basic Medical Sciences, Tianjin Medical University, Tianjin, China

**Keywords:** atherosclerosis, macrophage, VSMC, foam cells, crosstalk

## Abstract

**Introduction:**

Cardiovascular disease (CVD) caused by atherosclerosis (AS) remains the leading cause of mortality in developed countries. Understanding cellular heterogeneity within the inflammatory microenvironment is crucial for advancing disease management strategies. This study investigates the regulatory functions of distinct cell populations in AS pathogenesis, focusing on the interaction between vascular smooth muscle cell (VSMC)-derived ITLN1^+^ foam cells and SPP1^+^ FABP5^+^ macrophages.

**Methods:**

We employed single-cell RNA sequencing to characterize cell populations within AS plaques. Correlation analyses and the CellChat package were utilized to elucidate intercellular communication networks among various cell types. The functional roles of key subsets of macrophages and VSMCs were assessed using Gene Ontology (GO) and Kyoto Encyclopedia of Genes and Genomes (KEGG) pathway analyses. Pseudotime trajectory analysis was conducted to explore the dynamics of VSMC differentiation. Additionally, spatial transcriptomics analysis was used to demonstrate the physical interactions between different cell subpopulations.

**Results:**

We identified significant infiltration of macrophage clusters in AS, with SPP1^+^ FABP5^+^ macrophages being highly enriched in AS plaques. These macrophages were associated with lipid transport, storage, and cell migration pathways. A distinct subset of ITLN1^+^ foam cells derived from VSMCs exhibited robust expression of foam cell markers and lipid metabolism-related genes. Pseudotime trajectory analysis indicated that ITLN1^+^ foam cells represent a terminal stage of VSMC differentiation, characterized by elevated expression of genes linked to lipid synthesis and AS progression. Spatial transcriptomics and CellChat analysis revealed a significant interaction between ITLN1^+^ foam cells and SPP1^+^ FABP5^+^ macrophages, mediated by the MIF-(CD74 + CD44) and SPP1-CD44 ligand-receptor axes.

**Discussion:**

Our findings underscore the critical crosstalk between ITLN1^+^ foam cells and SPP1^+^ macrophages in promoting lipid accumulation and AS progression. Targeting this cell-cell interaction may offer new therapeutic avenues for managing atherosclerosis. Further validation of these mechanisms is necessary to develop effective immunotherapeutic strategies against AS.

## 1 Introduction

Atherosclerosis (AS), a chronic inflammatory condition, is the major cause of heart attacks and strokes (1, 2). Various treatment options have been explored, including lipid-lowering drugs, anti-platelet medications, nanomaterials, and other potential interventions (3, 4). However, the therapeutic efficacy of AS treatment is not ideal. Therefore, it is essential to investigate the cellular and molecular mechanisms involved in the progression of AS to identify potential targets for intervention. Previous research has indicated that macrophages and vascular smooth muscle cells (VSMCs) play essential roles in promoting plaque formation within arteries as well as inflammation within these plaques (5–7), thus making them promising targets for intervention.

The expression profile of VSMCs *in vivo* is highly complex and variable, contributing to the development of associated diseases through changes in cell phenotype (8). The plasticity and heterogeneity exhibited by VSMCs allow for their differentiation into various cell subsets (6, 8, 9). Single-cell RNA sequencing analysis revealed diverse subsets of VSMCs involved in AS, characterized by different gene expression patterns. For example, Tcf21 regulates VSMC phenotypic modulation and influences the number of fibromyocytes at the fibrous cap (10). In addition, differentiation into foam cells constitutes a canonical form of phenotypic modulation in VSMCs. The accumulation of lipid-rich foam cells within the arterial wall plays a pivotal role in the development and progression of AS lesions. Excessive accumulation of foam cells may result in necrosis within AS plaques (8, 11, 12). VSMCs are closely associated with foam cells, and research uncovered that the P2RY12/P2Y12 receptor facilitates the formation of VSMC-derived foam cells in advanced AS by regulating autophagy (13). Studies have confirmed that a significant proportion of foam cells in the human coronary arterial intima originates from smooth muscle cells. In the late stage of AS, a substantial number of cells expressing macrophage markers are derived from smooth muscle cells (14). However, the progress for AS therapies targeting foam cells has been constrained, due to an inadequate understanding of the underlying mechanisms (15).

Macrophages play an important role in the progression of AS. The plaque microenvironment affects macrophage polarization in different directions, and in addition to the two classic polarization types, other types of macrophage subsets also affect the development and regression of AS (5). SPP1^+^ macrophages, a unique subset of macrophages, are abundant in AS and play a pivotal role in the development and rupture of AS plaques (16). SPP1^+^ macrophages accumulate within the perivascular adipose tissue (PVAT) surrounding coronary arteries. This phenomenon contributes to fibrosis in PVAT and subsequently accelerates the narrowing of coronary arteries, facilitating the progression of AS (17). Recent reports demonstrate the crosstalk between SPP1^+^ macrophages and various cell types (18–23). Nevertheless, the precise involvement of SPP1^+^ macrophages in the progression of AS, their immunomodulatory function, and the intercellular communication within the plaque microenvironment remain to be fully elucidated.

The progression of the disease, however, does not depend solely on the autonomy of individual cells, but also on intercellular communication between different cell types. While the interaction between macrophages and VSMC-derived cells has been documented in AS research (24), the heterogeneity of VSMC subpopulations and variations in macrophage crosstalk remain poorly understood. Macrophages can induce the phenotypic transformation of VSMCs and accelerate the progression of AS through direct cell-cell contact or the secretion of relevant factors (25). Macrophages serve as an important source of foam cells, with the majority of current research focusing on macrophage-derived foam cells. However, the intercellular communication between macrophages, and VSMCs-derived foam cells remains underexplored. Unraveling the underlying mechanisms of this intricate interaction may contribute to the development of novel anti-AS therapies.

In this study, we identified the interaction between SPP1^+^ macrophages and ITLN1^+^ foam cells as a key facilitator of AS progression. Our findings highlight the strong correlation between VSMC-derived ITLN1^+^ foam cells and SPP1^+^ macrophage infiltration, resulting in increased lipogenesis and up-regulation of plaque-related genes. This interaction was further confirmed by spatial transcriptome analysis. Overall, our work reveals the intricate crosstalk between foam cells and macrophage subsets, underscoring their potential as therapeutic targets for AS treatment.

## 2 Result

### 2.1 Significant infiltration of macrophage clusters in AS

**Figure 1:**
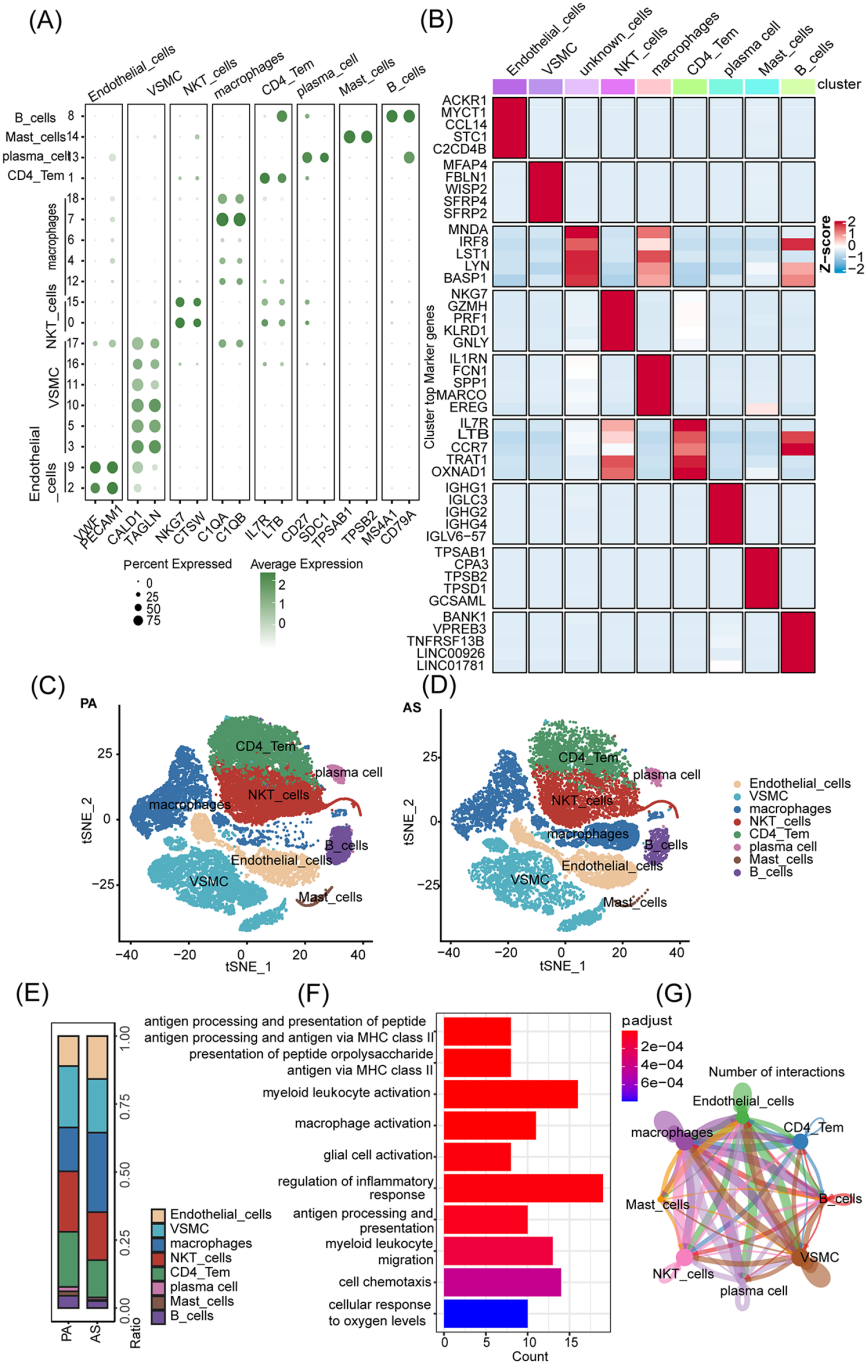
Significant infiltration of macrophage clusters in AS. **(A)** Cell type annotation. **(B)** Different cell clusters marker - average Heatmap display. **(C,D)** Plots of tSNE distribution in PA and AS. **(E)** The proportion of cell populations in PA and AS. **(F)** Performing a GO function enrichment analysis on the changing macrophage population. **(G)** Cell-cell interactions in AS and normal tissue samples.

In this study, we utilize the single-cell database GSE159677 (26) [comprising carotid atherosclerotic plaque (AS) and normal carotid tissue plaque area (PA)] for analysis of copy number variation and annotation of cell types. To accurately annotate cell types, we visualized the results of dimensionality reduction and clustering, as demonstrated in Figures 1A,B. There were 8 distinct cell types identified, including endothelial cells characterized by the expression of VWF (27) and PECAM1 (28), VSMCs distinguished by expression of CALD1 (29)and TAGLN (26, 30), NKT cells labeled with NKG7 (31) and CTSW (17) markers, macrophages defined by C1QA (21) and C1QB (32), CD4^+^ T cells identified by IL7R (33) and LTB (34) expression, plasma cells expressing CD27 (35) and SDC1 (36), mast cells labeled with TPSAB1 (37) and TPSB2 (38) markers, and B cells labeled by MS4A1 (39) and CD79A (40). Using Seurat and ggplot2 to draw the cell population proportional stack map of PA and AS cells tSNE distribution and cell population proportion map, we found that the macrophage cluster was obviously enriched in AS (Figures 1C–E). Gene Ontology (GO) functional analysis based on the differentially expressed genes between AS and PA revealed that this macrophage population was associated with macrophage activation, antigen presentation (MHC II), myeloid cell migration, and other pathways (Figure 1F). Cell-cell interactions in AS and normal tissue samples showed that macrophages have a strong interaction with a variety of cell types (Figure 1G).

### 2.2 SPP1^+^ FABP5^+^ macrophages are associated with the progression of AS

**Figure 2:**
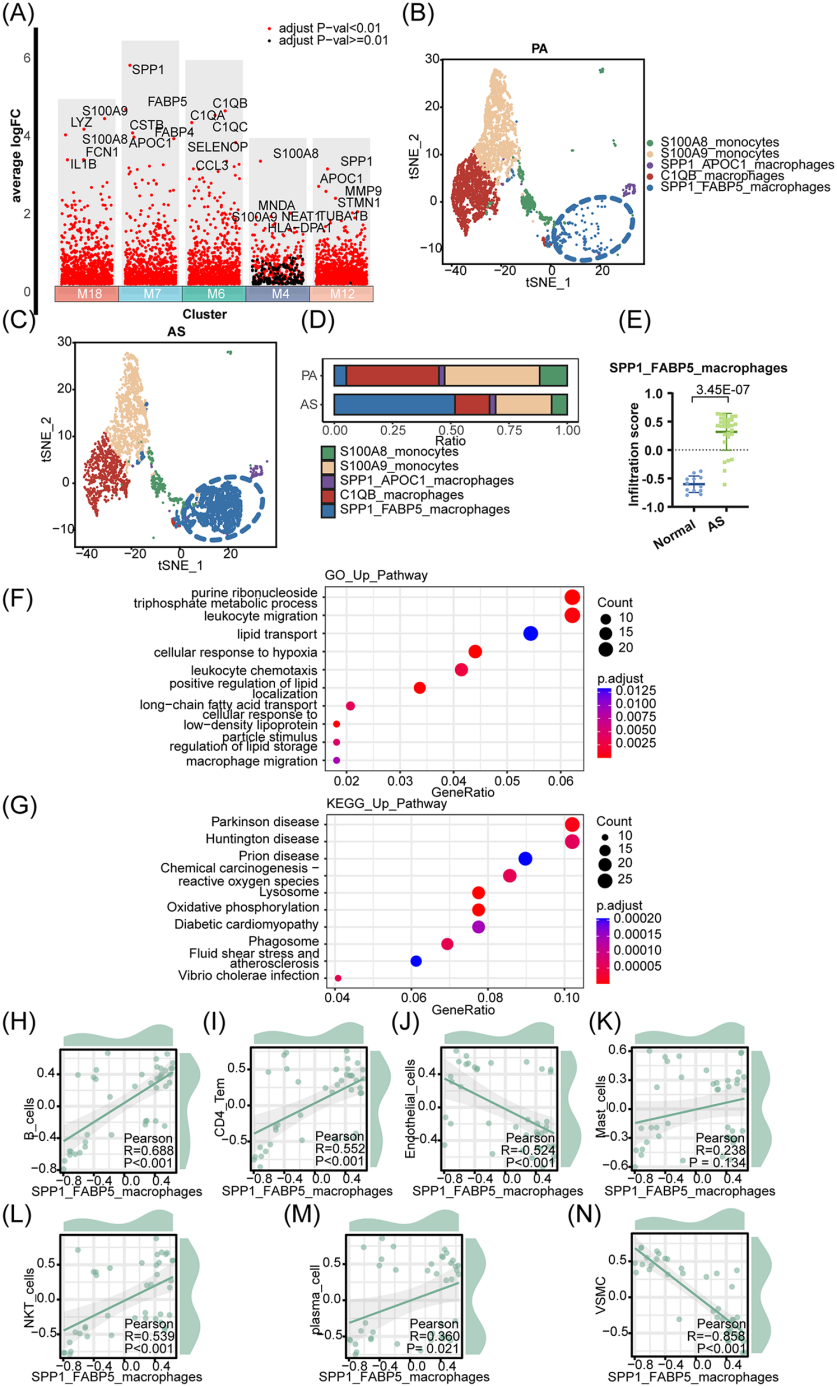
SPP1^+^ FABP5^+^ macrophages are associated with the progression of AS. **(A)** Volcano plot shows highly variable genes in each macrophage subpopulation. **(B,C)** Plots of tSNE distribution about macrophage subpopulations in PA and AS. **(D)** The proportion of macrophage subpopulations in PA and AS. **(E)** The top 25 highly variable characteristic genes of the SPP1^+^ FABP5^+^ macrophage population were used as the enrichment background gene set to analyze the enrichment differences of cell populations in the AS and PA groups. **(F,G)** The GO and KEGG functional enrichment analyses were conducted. **(H–N)** The correlation between SPP1^+^ FABP5^+^ macrophage and various cell types was examined using a scatter plot analysis.

To further classify the aforementioned macrophage populations and identify the macrophage subset with the most significant group differences, we generated multiple volcano plots to visualize the clusters of macrophage subsets (M18, M7, M6, M4, M12) (Figure 2A). Using t-SNE distribution and analysis of cell population proportions in PA and AS cells, we identified a significant enrichment of heterogeneous SPP1^+^ FABP5^+^ macrophages within the AS population, which has the largest proportion (Figures 2B–D). To gain deeper insights, we utilized the microarray dataset GSE100927 (41), which includes AS and PA samples, to extract the top 25 highly variable characteristic genes of SPP1^+^ FABP5^+^ macrophages as the enrichment background gene set. Single-sample gene set enrichment analysis (ssGSEA) showed the enrichment score of SPP1^+^ FABP5^+^ macrophages in the AS group (Figure 2E). To clarify the correlation between this macrophage subpopulation and the progression of AS disease, we conducted a functional analysis using GO and Kyoto Encyclopedia of Genes and Genomes (KEGG) pathway enrichment. The results showed that SPP1^+^ FABP5^+^ macrophages are characterized by lipid transport and storage, cell migration, and chemotaxis, all of which are associated with the pathogenesis of AS (Figures 2F,G). Furthermore, to identify cell populations significantly associated with SPP1^+^ FABP5^+^ macrophages, ssGSEA scores for each cell population were calculated from the microarray data, followed by correlation analysis using the corrplot and ggplot2 packages (Figures 2H–N). Interestingly, scatter plot analysis revealed the highest negative correlation between SPP1^+^ FABP5^+^ macrophages and VSMCs (Figure 2N). This finding contradicts our previous understanding that VSMC-macrophage interactions contribute to the progression of AS. We speculated that this negative correlation might be attributed to the inclusion of the PA cell population in our analysis, where VSMCs exhibit functions that are contrary to those of the SPP1^+^ FABP5^+^ macrophage population. Alternatively, VSMCs show varying functions at different stages of AS. The proliferation of VSMCs can stabilize AS during the early stage of AS (42, 43), which could also explain the negative correlation between VSMC and macrophages that we analyzed. Therefore, we then focus on VSMCs and identify pathogenic subpopulations that contribute to the progression of AS.

### 2.3 Identification and characterization of ITLN1^+^ foam cells derived from VSMCs in the progression of AS

**Figure 3:**
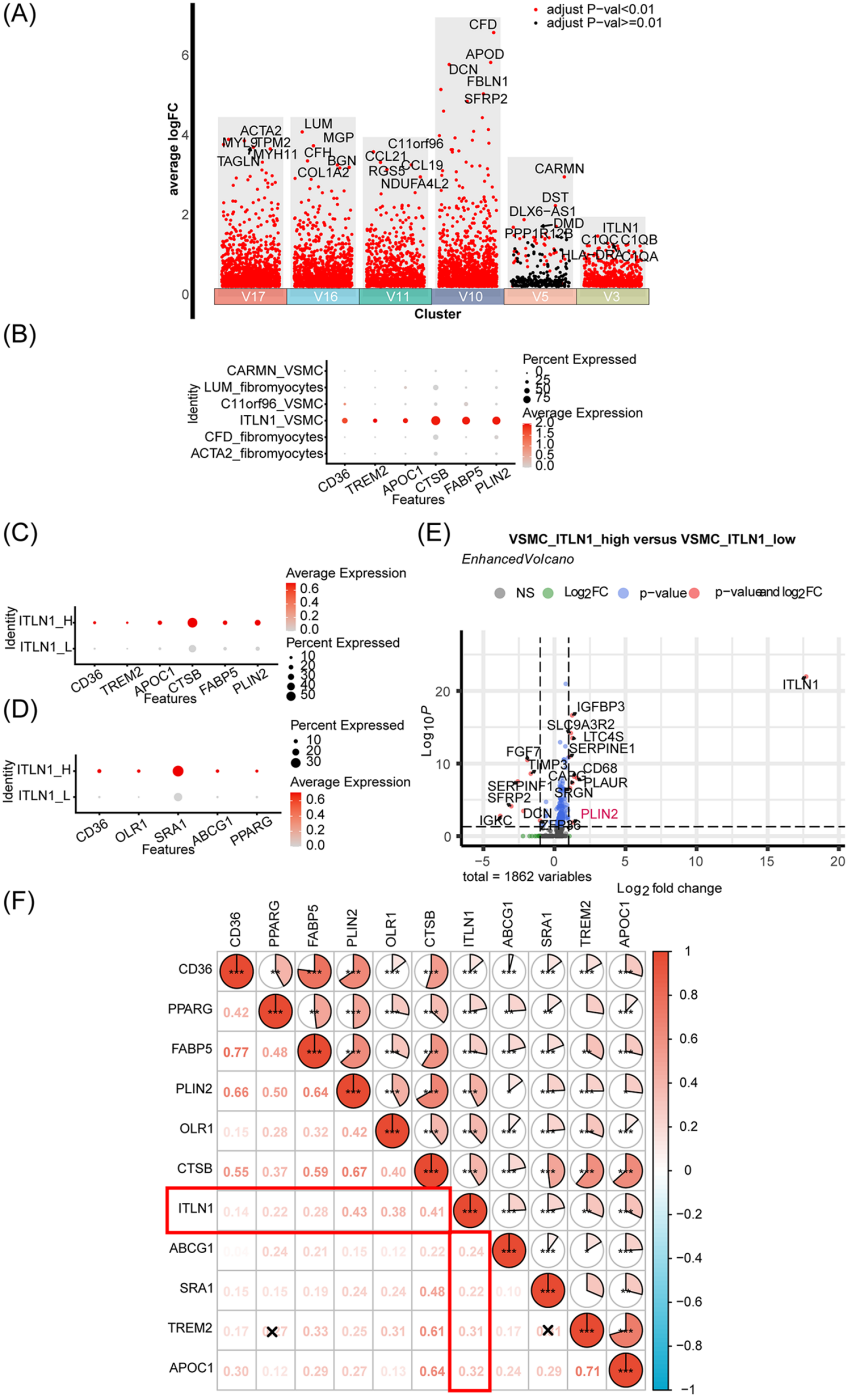
Identification and characterization of ITLN1^+^ foam cells derived from VSMCs in the progression of AS. **(A)** Volcano plot shows highly variable genes in each VSMC subpopulation. **(B)** Comparison of foam cell-related gene expression in different VSMC cell subsets. **(C,D)** Analysis of lipid and foam cell-related gene expression based on ITLN1 overexpression. **(E)** Volcano map was made based on VSMC_ITLN1_high and ITLN1_low differential genes. **(F)** The correlation pie chart illustrates the correlation of ITLN1 with lipids and foam cell markers.

As shown in Figure 3A, differential gene expression analysis was performed in the volcano map to identify the top 5 up-regulated markers in each VSMC subset (V17, V16, V11, V10, V5, V3). Notably, we identified a highly specific subset of ITLN1^+^ VSMCs (V3) that expressed traditional VSMC markers while also exhibiting macrophage-associated markers, such as C1QA (21) and C1QB (44). This suggests that these ITLN1^+^ VSMCs represent a distinct foam cell population derived from VSMCs, strongly associated with AS progression. To further investigate this hypothesis, we analyzed the expression of foam cell markers, including CD36, TREM2, APOC1, CTSB, FABP5, and PLIN2 (45–49), across distinct VSMC subpopulations (Figure 3B). Remarkably, ITLN1^+^ VSMCs exhibited robust expression of foam cell markers. Furthermore, the up-regulation of ITLN1 was found to enhance the expression of foam cell-related and lipid metabolism-related genes, such as CD36, OLR1, SRA1, ABCG1, and PPARG (26, 50–53) (Figures 3C,D). Additional analysis revealed differential gene expression between ITLN1_high and ITLN1_low VSMCs. Using the Enhanced Volcano package, we found that PLIN2, a key marker of foam cells, was significantly enriched in ITLN1_high VSMCs (Figure 3E). The pie chart in Figure 3F further underscores the strong correlation between ITLN1^+^ VSMCs and foam cell-related genes. In conclusion, our findings suggest that the ITLN1^+^ VSMC subset represents a foam cell population derived from VSMCs, highlighting its critical role in AS progression.

### 2.4 ITLN1^+^ foam cells associated with macrophages promote the progression of AS

**Figure 4:**
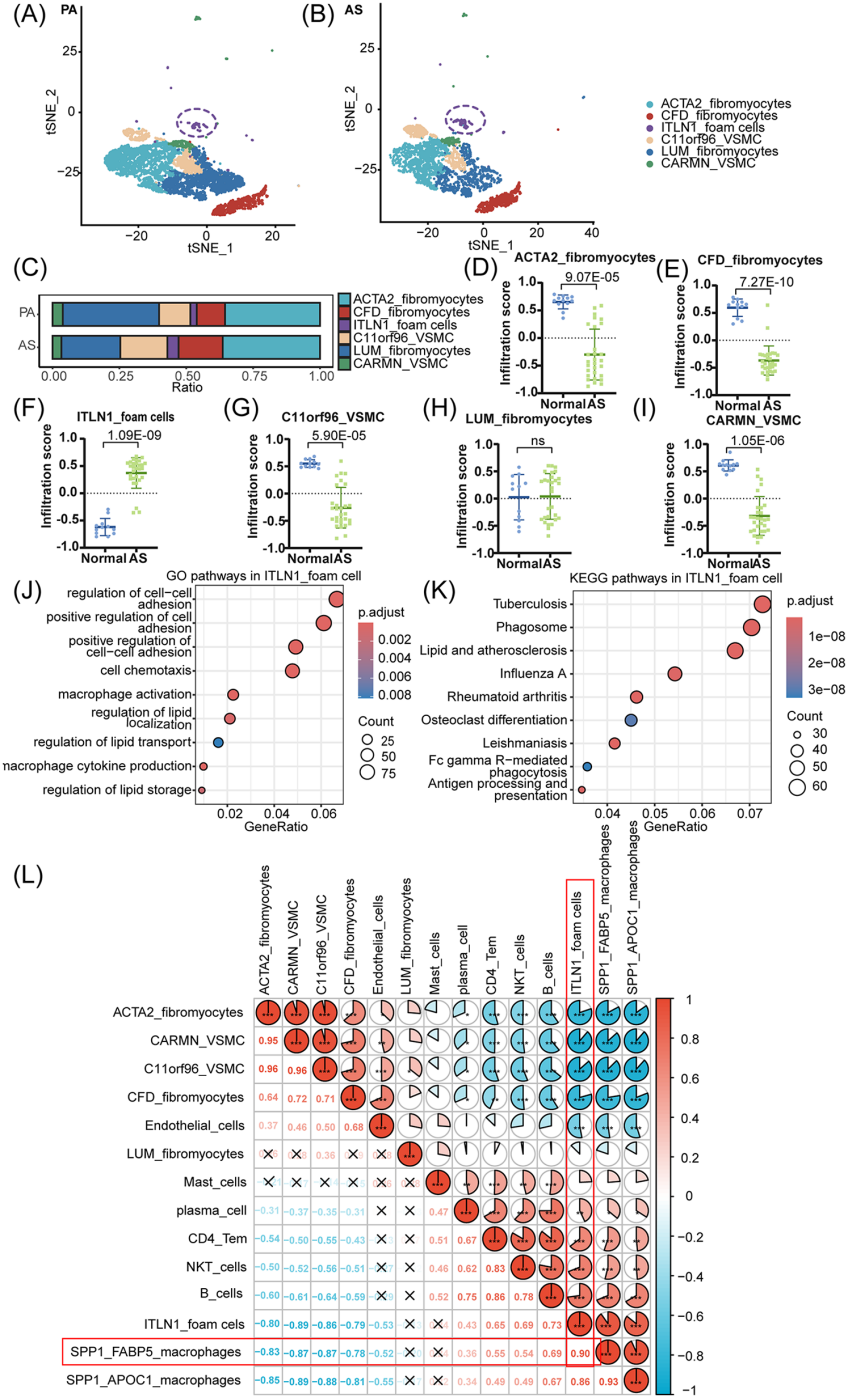
ITLN1^+^ foam cells associated with macrophages promote the progression of AS. **(A,B)** Plots of tSNE distribution about VSMC subpopulations in PA and AS. **(C)** The proportion of VSMC subpopulations in PA and AS. **(D-I)** The top 25 highly variable characteristic genes of 6 VSMC populations were used as the enrichment background gene set to analyze the enrichment differences of cell populations in the AS and PA groups. Among them, ITLN1^+^ foam cells are significantly enriched in AS. **(J,K)** The GO and KEGG functional enrichment analyses to elucidate the relationship between ITLN1^+^ foam cells and AS. **(L)** The correlation pie chart shows the correlation between SPP1^+^ FABP5^+^ macrophage and ITLN1^+^ VSMC subsets.

The tSNE distribution and cell population proportion analyses are performed to distinguish the differences between ITLN1^+^ foam cells and other VSMC subpopulations. The results revealed distinct cell subsets, including CFD^+^ fibromyocytes, C11orf96^+^ VSMCs, and ITLN1^+^ foam cells, with clear differentiation in the AS group compared to the PA group, as shown in Figures 4A–C. For each cell subset, 25 highly variable characteristic genes were selected as background gene sets for enrichment analysis. Notably, in Figures 4D–I, the gene signature of ITLN1^+^ foam cell displayed significant enrichment in AS compared to PA, highlighting a pronounced difference. Further functional analysis of ITLN1^+^ foam cells, as shown in Figures 4J–K, revealed their close association with macrophage activation, polarization, migration, and chemotaxis through GO and KEGG pathway enrichment analyses, implicating their pivotal role in the pathogenesis of AS. Additionally, as shown in Figure 4L, correlation analysis of cell populations was conducted using the same approach as described above. The resulting correlation pie chart revealed a strong relationship (*r* = 0.90) between ITLN1^+^ foam cells and SPP1^+^ FABP5^+^ macrophages, suggesting significant cross-talk interactions between these two cell clusters in AS.

### 2.5 ITLN1^+^ foam cells and SPP1^+^ FABP5^+^ macrophages interaction

**Figure 5:**
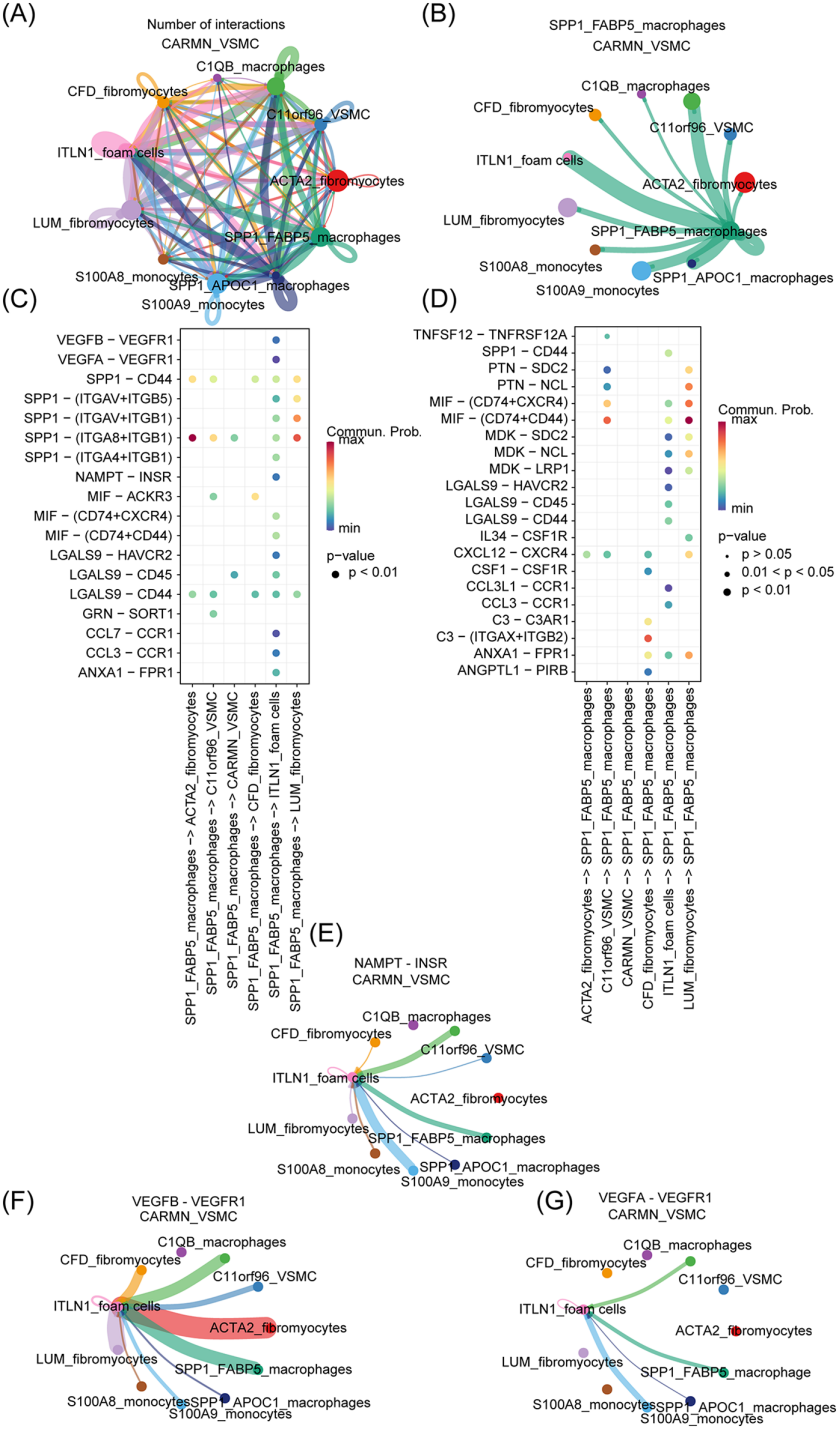
ITLN1^+^ foam cells and SPP1^+^ FABP5^+^ macrophage interaction. **(A)** The analysis of cell-cell interactions was employed to elucidate the ligand-receptor relationship between distinct subsets of cells. **(B)** The analysis of cell-cell interactions between SPP1^+^ FABP5^+^ macrophage and other cell clusters. **(C,D)** The investigation of cell-cell ligand-receptor pairs was conducted in tissue samples from both PA and AS. **(E–G)** The correlation between cell subsets and AS-related ligand receptors was analyzed.

To elucidate the interactions between these two cell clusters, further analysis was conducted. Based on the preprocessed t-SNE object, cell-cell interactions in AS and normal tissue samples were analyzed using the CellChat package (http://www.cellchat.org/), with the *CellChatDB human*. Intercellular ligand-receptor pairs were systematically explored in samples from both PA and AS tissues. A relatively dense network of ligand-receptor pairs was observed between ITLN1^+^ foam cells and SPP1^+^ FABP5^+^ macrophages, providing compelling evidence for their close intercellular communication, as shown in Figures 5A,B. Furthermore, Figures 5C,D visualizes the cell-cell communication network between SPP1^+^ FABP5^+^ macrophages and various cell populations derived from VSMCs, as mentioned above. SPP1^+^ FABP5_+_ macrophages exhibited a broader spectrum of ligandreceptor interactions with ITLN1^+^ foam cells, among which the MIF-(CD74 + CD44) and SPP1-CD44 ligand-receptor pairs were identified as key mediators facilitating the crosstalk between these distinct cell subsets. Additionally, as shown in Figures 5E–G, the CellChat analysis revealed the presence of ligand receptors, including VEGFB-VEGFR1, NAMPT-INSR, and VEGFA-VEGFR1 (54–56) which play a crucial role in AS, all participate in communication between these two types of cells, underscoring the importance of their interaction in AS.

### 2.6 ITLN1^+^ foam cells: key endpoint of pathogenic VSMC differentiation in AS

**Figure 6:**
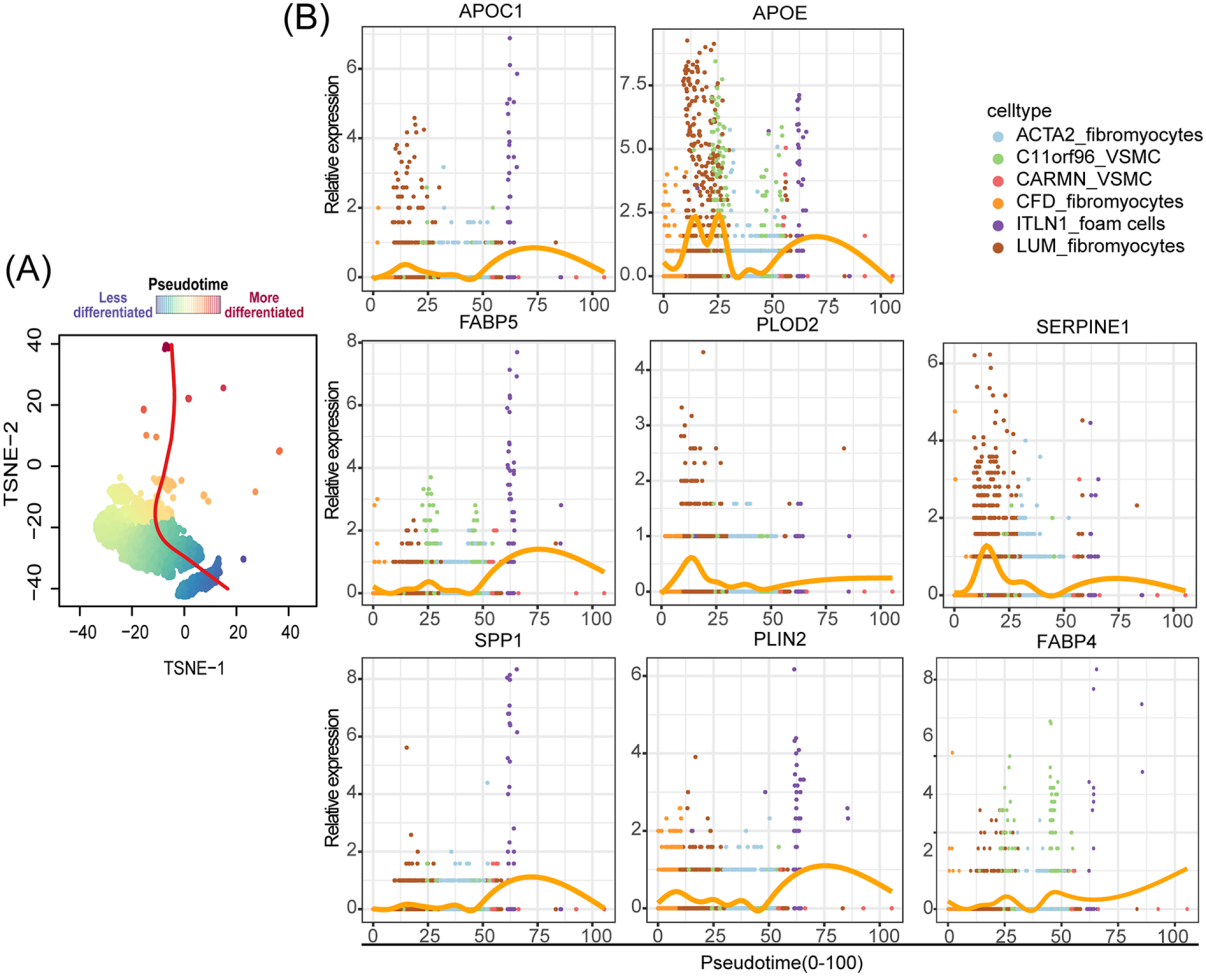
ITLN1^+^ foam cells: key endpoint of pathogenic VSMC differentiation in AS. **(A)** Schematic diagram of the cell pseudotime evolution trajectory, the gradient from blue to red signifies an increasing level of cellular differentiation. **(B)** Pseudotime kinetics of indicated genes from the root of the trajectory to the end.

To emphasize the role of ITLN1^+^ foam cells as a critical endpoint in the differentiation of VSMCs and their pivotal contribution to AS progression, we utilized scRNA-seq data to conduct pseudo-time trajectory analysis, which allowed us to examine the evolution of VSMC cells and the dynamic changes in pathogenic genes expression during differentiation, As the pseudo-time trajectory extended further backward, it signifies a more heterogeneous cellular state. By employing the Slingshot R package, we traced the evolutionary dynamics of single-cell transcriptomes. As shown in Figure 6A, the extension of the evolutionary curve reflects the progressive differentiation of VSMCs. The analysis considered six VSMC subpopulations as potential differentiation trajectories, and we selected lipid metabolism and AS-related genes, including APOC1, APOEA, FABP5, PLOD2, SERPINE1, SPP1, PLIN2, and FABP4 (57–63), to analyze the dynamic changes in their expression levels along the evolutionary trajectory. Dimensionality reduction was performed using the reducedDims function and differentially expressed genes (DEGs) along pseudotime were identified with the tradeSeq package. Marker gene expression trends were visualized using pseudotemporal heatmaps generated with the plotGeneCount function. As shown in Figure 6B, ITLN1^+^ foam cells were found to represent a highly evolved pathogenic VSMC subpopulation, characterized by elevated expression of genes linked to lipid synthesis and AS progression. Among the genes we selected, the expression levels of SPP1 and FABP5 are significantly higher in the evolutionary node of ITLN1^+^ foam cells compared to other pseudo-temporal evolutionary nodes, providing support for our hypothesis regarding the interaction between SPP1^+^ FABP5^+^ macrophages and ITLN1^+^ foam cells. These findings underscore the highly differentiated state of ITLN1^+^ foam cells and their significant role in lipid accumulation and plaque formation, highlighting their critical involvement in AS pathology.

### 2.7 Spatial transcriptomics reveals physical interactions between ITLN1^+^ foam cells and SPP1^+^ macrophages

**Figure 7:**
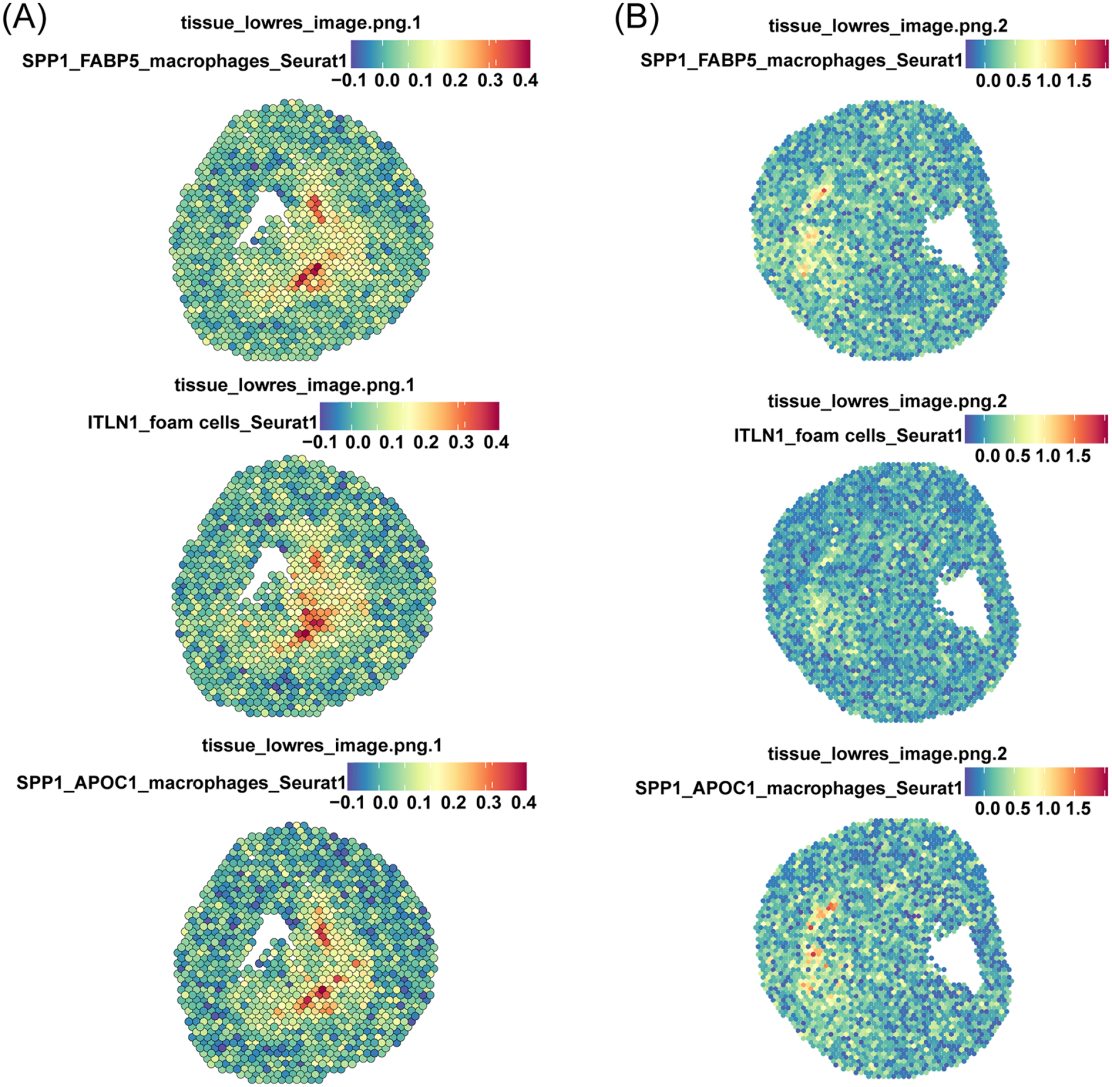
Spatial transcriptomics reveals physical interactions between ITLN1^+^ foam cells and SPP1^+^ macrophages. Spatial transcriptomics datasets derived from two tissue sections of AS patients as shown in **(A,B)**, respectively.

**Figure 8:**
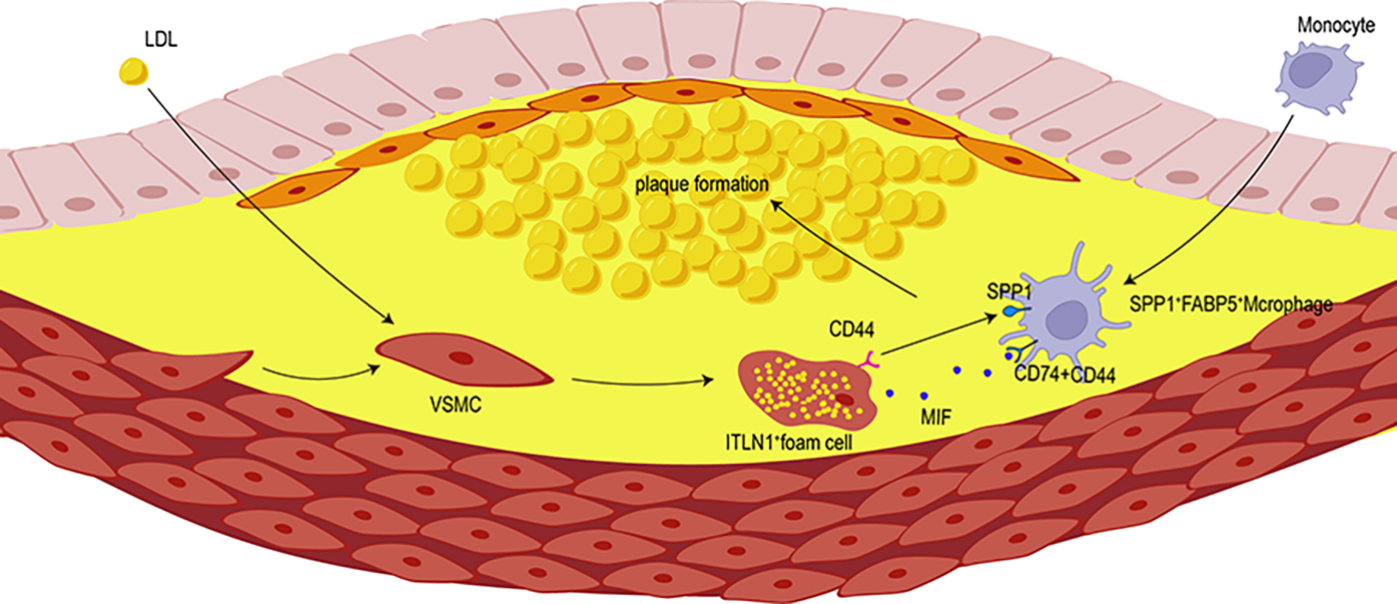
The interactions between SPP1^+^ macrophages and VSMCs facilitate the transformation of VSMCS into ITLN1^+^ foam cells. SPP1^+^ macrophages promote the recruitment of ITLN1^+^ foam cells to the plaque area, thereby accelerating plaque formation.

The spatial co-localization of ITLN1^+^ foam cells and SPP1^+^ FABP5^+^ macrophages was analyzed using spatial transcriptomics (ST) datasets GSE243179 (64) (Figure 7A) and GSE159677 (26) (Figure 7B), derived from tissue sections of AS patients. These datasets provided high-resolution localization of specific gene expressions. We analyzed the gene-spot matrix and normalized the data, followed by visualizing the cell clusters on the slices. The analysis revealed a high degree of spatial similarity among ITLN1^+^ foam cells, SPP1^+^ FABP5^+^ macrophages, and SPP1^+^ APOC1^+^ macrophages. This finding was unexpected, as the SPP1^+^ APOC1^+^ macrophage subpopulation was previously identified as significantly smaller than SPP1^+^ FABP5^+^ macrophages. Moreover, the correlation heatmap (Figure 4L) demonstrated that SPP1^+^ APOC1^+^ macrophages had a lower correlation with ITLN1^+^ foam cells (*r* = 0.83) compared to SPP1^+^ FABP5^+^ macrophages (*r* = 0.90). As a result, SPP1^+^ APOC1^+^ macrophages were not emphasized in the analysis. Nevertheless, the significant correlation suggests that SPP1 may play a critical role as a macrophage-related gene. Collectively, the high expression of SPP1 likely enables macrophage subpopulations to interact with ITLN1^+^ foam cells, potentially contributing to the progression of AS (Figure 8).

## 3 Discussion

AS is a chronic inflammatory vascular disease characterized by intimal thickening, smooth muscle cell proliferation, lipid accumulation, and plaque formation. Despite recent advances, clinical treatments for AS remain limited, and its underlying mechanisms are still not fully understood. Current research highlights the indispensable role of macrophages in the development of AS, including their involvement in inflammation, intercellular communication, plaque development, and rupture (5). Macrophages engage in a complex communication network with other cell types, collectively driving the progression of AS (64, 65). In this study, we investigated the intricate interactions between VSMC-derived foam cells and macrophages, highlighting the critical crosstalk between ITLN1^+^ foam cells and SPP1^+^ macrophages, which offered comprehensive insights into the pathogenesis of AS.

Phosphoprotein 1 (SPP1) has emerged as a potential diagnostic biomarker for AS and is closely associated with lipid synthesis (61, 66, 67). It is significantly expressed in pathological macrophages, where SPP1^+^ macrophages play a critical role in the development and progression of AS (16). In our study, single-cell profiling revealed that pathways related to lipid transport, storage, and cell migration were enriched in SPP1^+^ macrophages. Studies have demonstrated that there is a crosstalk between SPP1^+^ macrophages and multiple cell subtypes (68–70). CellChat analysis reveals robust interactions between macrophages and VSMC. Given that VSMC transdifferentiation contributes to plaque complexity and vulnerability, our study identifies key VSMC subtypes involved in AS progression, particularly ITLN1^+^ foam cells. ITLN1, encoding intelectin-1 associated with obesity and hypertension, may drive the processes in AS (26, 71). Notably, approximately 50% of foam cells in AS lesions originate from VSMCs (14, 72), contributing to lipid accumulation and inflammation, thereby promoting the formation of AS (73). Therefore, the interaction between SPP1^+^ macrophages and ITLN1^+^ foam cells was further analyzed. Correlation analysis and spatial transcriptomics revealed a significant positive relationship between the two cells, which co-localized in plaque centers and promoted AS progression. Trajectory analysis suggested that ITLN1^+^ foam cells represent the terminal stage of VSMC differentiation. Interestingly, genes related to lipid production and SPP1 gradually increased during the evolution of VSMC towards ITLN1^+^ foam cells, which exacerbated lipid deposition and plaque formation. These findings indicate that pathological differentiation of VSMCs into ITLN1^+^ foam cells promotes the development of AS, whereas targeting ITLN1^+^ foam cells may alter the progression of AS.

Single-cell sequencing has demonstrated that macrophages secrete IL-1β in response to oxidized low-density lipoprotein (ox-LDL), which influences the formation of VSMC-derived foam cells (74). Foam cell formation in VSMC occurs after macrophage infiltration into the plaque (73, 75). M2-like macrophages within AS plaques promote the phenotypic conversion of VSMCs, with foam cell formation being one possible cell fate (3). These findings suggest that macrophages may recruit VSMC to plaques and induce foam cell formation through cytokine secretion or direct cell-cell contact. However, research on foam cells remains limited, and there is a gap in single-cell transcriptomic studies that focus on interactions between macrophage and VSMC-derived foam cells. In our study, SPP1^+^ macrophages not only increase in AS, but also recruit VSMCs to the plaque center through various signaling pathways, such as SPP1-CD44, and MIF-(CD74 + CD44). The SPP1-CD44 and MIF-(CD74 + CD44) axis facilitate the establishment of a sophisticated cellular communication network among macrophages and other cell subsets (76–79). Targeting the CD44 receptor disrupts the interaction between macrophages and VSMCs, resulting in the polarization of macrophages towards an inflammatory phenotype and influencing VSMC phenotypic switching (80). These findings suggest that SPP1^+^ macrophages mediated the recruitment of VSMCs via SPP1 signaling, promoting VSMC differentiation into foam cells, and enhanced lipid biosynthesis and transport, thereby exacerbating AS. Our results highlight the dynamic crosstalk between macrophages and foam cells as part of a complex signaling network within the AS microenvironment. This interaction plays a crucial role in advancing AS, and targeting this network may offer potential therapeutic strategies.

Spatial transcriptome analysis revealed that ITLN1^+^ foam cells, SPP1^+^ FABP5^+^ macrophages, and SPP1^+^ APOC1^+^ macrophages share similar spatial distributions. We focused on SPP1^+^ FABP5^+^ macrophages due to their larger population and stronger association with ITLN1^+^ foam cells compared to SPP1^+^ APOC1^+^ macrophages. The spatial physical interactions between SPP1^+^ FABP5^+^ macrophage and ITLN1^+^ foam cells suggest a close interaction between these two cell types. Both SPP1 and FABP5 are closely involved in lipid metabolism and macrophage function. The delineation of SPP1^+^ FABP5^+^ macrophages provides a biological context for understanding foam cell formation and the crosstalk between the two cell types.

Although we revealed the intercellular interaction of macrophages and foam cells in promoting the development of AS, it is important to acknowledge the limitations of this study, particularly regarding the validation of sequencing data. Although we utilized various scRNA-seq and spatial transcriptomics to analyze cellular interactions, these findings still require further validation through additional experiments. Future studies should aim at expanding sample sizes and employing diverse experimental platforms to enhance the understanding of these cell populations and their interaction mechanisms, which may offer new insights for developing interventions in AS.

Overall, we identified a novel subpopulation of SPP1^+^ macrophages and analyzed their interactions with ITLN1^+^ foam cells. This study provides evidence of the intricate relationship between macrophages and foam cells, providing a new perspective on the progression of AS.

## 4 Methods

### 4.1 Data collection

The information regarding the dataset is illustrated in tabular form, as depicted in Table 1.

**Table 1:** The information about the dataset.

Data set	Database of data	Type of data	Detailed information
GSE243179	GEO	Spatial transcriptomics	Sample 1 (coronary sample with plaque erosion from a 42 year-old white female) Sample 2 (coronary sample with plaque erosion from a 46 year-old white female)
GSE159677	GEO	Single-cell transcriptomics	Calcified atherosclerotic core (AC) plaques and patient-matched proximal adjacent (PA) portions of carotid artery were collected from three patients.
GSE100927	GEO	Bulk RNA transcriptomics	Control carotid (*n* = 12) AS carotid (*n* = 29)

### 4.2 Single-cell copy number variation analysis and cell type annotation

In order to identify different cell types, the Seurat package was used to remove low-quality cells, including those with more than 8,000 detected genes or fewer than 200, as well as those with mitochondrial gene expression exceeding 20% of total expression. After filtration, data normalization was performed using the NormalizeData function within the Seurat package, with a scaling factor of 10,000. Next, the FindVariableFeatures function was used to identify highly variable genes, followed by RunPCA for dimensionality reduction on the normalized dataset. Clustering analysis was then performed using the FindNeighbors and FindClusters functions. Dimensionality reduction and clustering results were visualized using UMAP and t-SNE techniques. Cell types were annotated using the classical marker genes of known cell types in combination with the SingleR packages. Marker genes for each cluster were defined based on their expression levels, where these genes were expressed in more than 30% of cells and had an average expression value exceeding 0.5.

### 4.3 Differential analysis of cell population enrichment

To perform enrichment and analysis of distinct cell populations, the Seurat package was used to preprocess the scRNA-seq data and conduct cell clustering. The metadata of cells was extracted from the processed Seurat object, including the sample ID, group, cell types, and cell clusters. Using the dplyr package, cells were grouped, and the number of different cell populations in each sample was calculated. Subsequently, the proportions of each cell population were determined. The results of the cell composition proportions for each group were visualized using the ggplot2 package.

The dataset GSE100927 containing AS and PA data was used to identify the top 25 most variable feature genes from each cell subpopulation, thereby creating an enriched background gene set. The GSVA package was employed to perform single-sample gene set enrichment analysis (ssGSEA) on this dataset and calculate the ssGSEA score for the dataset. After calculating the score, the enrichment scores of these clusters in the AS and PA groups were compared to measure the difference in the proportion of specific cell populations in these two groups.

### 4.4 Functional enrichment analysis

To clarify the biological roles of cell subpopulations, we conducted analyses using gene ontology (GO) and the Kyoto Encyclopedia of Genes and Genomes (KEGG), establishing a significance threshold of *P* < 0.05 to identify relevant pathways.

### 4.5 Analysis of cellular interactions

To assess the activity of various ligand-receptor pairs across distinct cell populations and the enrichment of signaling pathways, the CellChat analysis was conducted. Based on the preprocessed tSNE object, cell-cell interactions in AS and normal tissue samples were analyzed by running the CellChat package (http://www.cellchat.org/), with the interactive ligand-receptor database set as CellChatDB human.

### 4.6 Analysis of pseudo-temporal trajectories in scRNA-Seq datasets

We performed a pseudo-temporal trajectory analysis on scRNA-seq datasets using Slingshot R package to investigate the dynamic changes in gene expression in different cell states. Slingshot enables the inference of developmental trajectories by identifying continuous, ordered cell states, and ordering cells along a pseudotime axis that represents their progression through a biological process. Using the reducedDims function in Slingshot, we apply methods such as PCA, t-SNE to project the cells into a lower-dimensional space, facilitating the identification of distinct cell groups. These projections were then processed by Slingsho to infer the pseudotime trajectory. To identify the differentially expressed genes (DEGs) along the pseudotime continuum, we use the tradeSeq package, which is designed for modeling gene expression along continuous trajectories. To better understand how gene expression changes over pseudotime, we use the plotGeneCount function to create heatmaps visualizing the expression of marker genes or DEGs across the pseudotime continuum.

### 4.7 Analysis of spatial transcriptomics data

In order to demonstrate the spatial interactions between cell subpopulations, the spatial transcriptomics (ST) datasets GSE243179 and GSE159677 from two AS patients were incorporated. The Seurat package was used to process both ST and Visium sample data, generating gene-spot matrices. Gene expression data were normalized using the SCTransform function. Dimensionality reduction and clustering were performed using principal component analysis (PCA), followed by mapping cell clusters onto tissue images with the SpatialDimPlot function. Signature scoring was conducted using the AddMetaData function in the Seurat package, based on scRNA-seq or ST signatures, with default parameters. Finally, spatial feature expression plots were generated using the SpatialFeaturePlot function in the Seurat package.

## Data Availability

Publicly available datasets were analyzed in this study. These datasets have been deposited at the Gene Expression Omnibus (GEO) database, including spatial transcriptomics dataset under accession number GSE243179 (https://www.ncbi.nlm.nih.gov/geo/query/acc.cgi?acc=GSE243179), single-cell transcriptomics under accession number GSE159677 (https://www.ncbi.nlm.nih.gov/geo/query/acc. cgi?acc=GSE159677), and bulk transcriptomics under accession number GSE100927.
